# Serum and Nasal Lavage Fluid Eosinophil‐Derived Neurotoxin Levels in Clinically Defined Asthma Phenotypes

**DOI:** 10.1002/clt2.70183

**Published:** 2026-06-23

**Authors:** Saliha Selin Özuygur Ermis, Carina Malmhäll, Magnus P. Borres, Robert Movérare, Daniil Lisik, Reshed Abohalaka, Selin Ercan, Susanne Schmeisser, Rani Basna, Roxana Mincheva, Göran Wennergren, Jan Lötvall, Linda Ekerljung, Madeleine Rådinger, Hannu Kankaanranta, Bright I. Nwaru

**Affiliations:** ^1^ Krefting Research Centre Institute of Medicine Sahlgrenska Academy University of Gothenburg Gothenburg Sweden; ^2^ Department of Women's and Children's Health Uppsala University Uppsala Sweden; ^3^ Thermo Fisher Scientific Uppsala Sweden; ^4^ Department of Medical Sciences: Respiratory, Allergy and Sleep Research Uppsala University Uppsala Sweden; ^5^ Department of Public Health and Clinical Medicine, Section of Sustainable Health The OLIN Unit Umeå University Umeå Sweden; ^6^ Department of Clinical Immunology Sahlgrenska University Hospital Gothenburg Sweden; ^7^ Division of Geriatric Medicine, Department of Clinical Sciences in Malmö Lund University Malmö Sweden; ^8^ Department of Paediatrics, Queen Silvia Children's Hospital Sahlgrenska University Hospital University of Gothenburg Gothenburg Sweden; ^9^ Department of Respiratory Medicine and Allergology Sahlgrenska University Hospital Gothenburg Sweden; ^10^ Tampere University Respiratory Research Group Faculty of Medicine and Health Technology Tampere University Tampere Finland; ^11^ Diagnostic Centre The Wellbeing Services County of South Ostrobothnia Seinäjoki Finland; ^12^ Wallenberg Centre for Molecular and Translational Medicine University of Gothenburg Gothenburg Sweden

**Keywords:** adult, asthma, biomarker, eosinophil, eosinophil‐derived neurotoxin

## Abstract

**Background:**

As a surrogate for eosinophilic activation, eosinophil‐derived neurotoxin (EDN) is a potential clinical biomarker. However, EDN levels and discriminatory ability in different asthma phenotypes are unknown. We quantified serum and nasal lavage fluid (NLF) EDN levels and assessed the potential to differentiate clinically defined asthma phenotypes in an adult‐representative sample.

**Methods:**

A total of 1499 serum and 386 NLF samples from individuals with current asthma obtained from the West Sweden Asthma Study were analyzed for EDN. Eosinophilic asthma was defined as blood eosinophil count of ≥ 300 cells/mm^3^, T2‐high asthma was defined as blood eosinophil count of ≥ 300 cells/mm^3^ or fractional exhaled nitric oxide (FeNO) levels of ≥ 25 ppb. Other asthma phenotypes were defined based on presence of atopy, chronic rhinosinusitis (CRS), nasal polyposis, and obesity.

**Results:**

Subjects with eosinophilic asthma had higher serum and NLF EDN levels than those with non‐eosinophilic asthma. In ROC analyses, serum EDN provided excellent discrimination between eosinophilic and non‐eosinophilic asthma (area under curve [AUC] = 0.84, 95% CI = 0.82–0.86); the corresponding AUC for NLF EDN was 0.67 (95% CI = 0.62–0.73). Serum and NLF EDN were higher in atopic asthma, T2‐high asthma, and asthma with nasal polyposis compared to their counterparts, but not in asthma with CRS. The highest absolute values of serum and NLF EDN were observed in the high eosinophil + FeNO group, followed by the high‐eosinophil‐only and high‐FeNO‐only groups.

**Conclusion:**

Both serum and NLF EDN were higher in those with eosinophilic compared to non‐eosinophilic asthma. However, only serum EDN appears to distinguish eosinophilic asthma in ROC analyses.

## 1 Introduction

Asthma is a common airway disease characterized by chronic airway inflammation and airway hyperresponsiveness [1]. Disease course and clinical outcomes vary among individuals with different characteristics [2, 3]. Therefore, the need to accurately characterize asthma phenotypes has increasingly become pertinent [2, 3].

Clinically useful biomarkers are needed for asthma phenotyping [4]. Eosinophil‐derived neurotoxin (EDN), an eosinophil granule protein, has recently emerged as one of such potential biomarkers, with the ability to reflect eosinophilic activation [5]. In previous studies, individuals with asthma had higher EDN levels than those without asthma [6, 7]. However, only a few studies have quantified EDN levels in adult asthma phenotypes so far [8, 9]. In one such study, serum EDN levels were higher in adults with eosinophilic asthma compared to those with mixed granulocytic and neutrophilic asthma [8]. Serum EDN was also able to discriminate adults with asthma and high blood eosinophil levels [9]. Previous literature has also shown that children with atopic asthma have higher serum EDN levels; however, these findings have not yet been confirmed in a representative adult sample [10, 11].

In addition to eosinophilic and atopic asthma, EDN warrants further investigation as a potential biomarker for coexistence of chronic rhinosinusitis (CRS) and asthma, given the underlying eosinophilic inflammation in the pathogenesis of both diseases [12]. Previously, presence of CRS has been associated with high eosinophil count in those with severe asthma [13]; however, EDN levels have not been investigated in the same context. Considering that there is a strong association between asthma and CRS, particularly with nasal polyposis, EDN could be a potential biomarker to identify these specific patient subgroups [14, 15, 16, 17, 18].

While evidence elucidating the role of EDN in asthma phenotyping is sparse, to the best of our knowledge, no previous population‐based study has determined levels of serum and nasal lavage fluid (NLF) EDN in different asthma phenotypes and whether EDN can potentially discriminate between different asthma phenotypes. The aim of the current study was to (i) quantify serum and NLF EDN levels in different clinically defined asthma phenotypes in a general population‐representative adult sample, and (ii) to determine the ability of serum and NLF EDN to differentiate between different asthma phenotypes in such a population.

## 2 Methods

### 2.1 Ethics Approval

Ethical approval for West Sweden Asthma Study (WSAS) was acquired from the Swedish Ethical Review Authority and Ethics Committee of the University of Gothenburg (034–08, 593–08, 052/16, 906/16). All participants gave written informed consent to participate in the current study.

### 2.2 Study Population

Study participants were obtained from WSAS, an ongoing population‐based representative longitudinal cohort study. Residents of western Sweden (Västra Götaland region) who aged between 16 and 75 years old, were randomly invited to participate a postal questionnaire (Supporting Information S1: Figures E1 and E2). Postal questionnaires were sent to a random population of 30,000 subjects in 2008, of which 18,087 (62%) participated. Of the responders, 2006 (a random sample and asthma sample) individuals participated in a detailed clinical investigation. Incident asthma cases from the follow‐up of the first investigation were also included. In 2016, another non‐overlapping random sample of adults was invited to complete a similar postal questionnaire. Of 50,000 subjects invited, 24,534 (50%) participated. A random sample and asthma sample were invited for clinical investigations, of which 953 individuals had been examined up until the onset of the COVID‐19 pandemic. Serum EDN analysis was performed in subjects with available serum samples from both clinical investigations (*n* = 2939), while NLF EDN analysis was only performed in the first clinical investigation due to sample and cell count availability (*n* = 878) [19]. Among them, individuals with current asthma were included in the present study (Supporting Information S1: Figure E1 and E2). Detailed study protocol and characteristics of the study have been previously reported [19, 20, 21].

### 2.3 Serum and NLF EDN Measurements

EDN analysis in serum and NLF samples was conducted using the ImmunoCAP EDN kit (research use only) in line with manufacturer instructions (Phadia AB, Thermo Fisher Scientific, Uppsala, Sweden). The standard operating procedures for serum and NLF samples have been described previously [19]. The distribution of serum and NLF EDN levels, and related factors to high EDN levels were detailedly presented in a previous paper [19].

### 2.4 Blood Eosinophil Count, NLF Cell Percentage, and Fractional Exhaled Nitric Oxide (FeNO) Measurement

As previously described, blood samples were collected into EDTA tubes for complete blood count and transferred to the Clinical Chemistry Laboratory of Sahlgrenska University Hospital [22]. NLF eosinophil percentage were calculated within all cell types. Lastly, FeNO measurement was performed at 50 mL/s flow rate by NIOX VERO (Aerocrine, Morrisville, NC) device [22].

### 2.5 Definition of Current Asthma and Asthma Phenotypes

*Current asthma* was defined as the presence of physician‐diagnosed/ever asthma and at least one of: (i) self‐reported symptoms (wheezing and/or attacks of shortness of breath during the last 12 months), (ii) asthma medication use during the past 12 months, and (iii) a positive bronchodilator response at the clinical visit. Of the 2939 subjects whose samples were analyzed for serum EDN levels, 1499 (51%) reported current asthma (Supporting Information S1: Figure E1). Among those who had available NLF EDN analysis (*n* = 878), 386 (44%) reported current asthma (Supporting Information S1: Figure E2).

The following asthma phenotypes were defined among those with current asthma:

#### 2.5.1 Eosinophilic Asthma

High blood eosinophil count (≥ 300 cells/mm^3^).

#### 2.5.2 Atopic Asthma

Allergic sensitization (positive skin prick test [≥ 3 mm] or specific immunoglobulin E (IgE) positivity [≥ 0.35 kU_A_/L]) to common aeroallergens (please see Supporting Information S1 for details).

#### 2.5.3 Atopic Eosinophilic Asthma

High blood eosinophil count (≥ 300 cells/mm^3^) and atopy. Those with high blood eosinophil count without atopy was classified as *“non‐atopic eosinophilic asthma”.*

#### 2.5.4 Type 2 (T2)‐High Asthma

Either high blood eosinophil count (≥ 300 cells/mm^3^) or high FeNO (≥ 25 ppb) or both. In addition, we performed sensitivity analysis for T2‐high asthma (*T2‐high asthma with atopy*) by a broadened set of criteria based on presence of any of the following: high blood eosinophil count (≥ 300 cells/mm^3^) or high FeNO (≥ 25 ppb) or presence of atopy.

Subgroups were also formed for (i) high‐eosinophil‐only (blood eosinophil count ≥ 300 cells/mm^3^ and FeNO < 25 ppb), (ii) high‐FeNO‐only (FeNO ≥ 25 ppb and blood eosinophil count < 300 cells/mm^3^), (iii) high eosinophil + FeNO (blood eosinophil count ≥ 300 cells/mm^3^ and FeNO ≥ 25 ppb), and (iv) low eosinophil + FeNO (blood eosinophil count < 300 cells/mm^3^ and FeNO < 25 ppb).

#### 2.5.5 Asthma With Chronic Rhinosinusitis (CRS)

Those with self‐reported CRS based on affirmative response to at least two of the following symptoms, present for more than 12 weeks in the past year: nasal blockage, nasal secretion, facial pain/pressure, and smell impairment. Further, at least one of the self‐reported symptoms needed to be either nasal blockage or nasal secretion [23, 24].

#### 2.5.6 Asthma With Nasal Polyposis

Self‐reported nasal polyposis.

#### 2.5.7 Asthma With CRS and Nasal Polyposis (CRSwNP)

Self‐reported CRS and self‐reported nasal polyposis. Those with self‐reported CRS, without self‐reported nasal polyposis were defined as *“asthma with CRS without nasal polyposis (CRSsNP)”*.

#### 2.5.8 Obese Asthma

Those with body mass index (≥ 30 kg/m^2^).

### 2.6 Statistical Analysis

NLF EDN levels below the lower limit of detection as well as missing values for blood eosinophil count and FeNO levels were imputed (Please see Supporting Information S1 for details). Details of the employed imputation methods have been previously described [19]. Non‐parametric tests (Mann‐Whitney‐*U* or Kruskal Wallis) was used to compare EDN levels in asthma phenotypes due to non‐normal distribution of the serum and NLF EDN levels. Receiver operating characteristic (ROC) analysis was used and area under curve (AUC) was calculated to evaluate the ability of EDN to discriminate between asthma phenotypes. Optimal cut‐off values based on Youden index, sensitivity, specificity, positive predictive value, and negative predictive value were calculated using pROC package [25]. AUC levels equal or higher than 0.9 were determined as *outstanding discrimination,* < 0.9–≥ 0.8 *excellent discrimination*, < 0.8 to ≥ 0.7 as *acceptable discrimination*, < 0.7 to > 0.5 as *poor discrimination*, and 0.5 as *no discrimination* based on previous literature [26]. Logistic regression analysis was performed to evaluate the association between asthma phenotypes and EDN levels. EDN levels were divided into 4 quartiles and 1st quartile was the referent group. A two‐sided *p*‐value was set at < 0.05 to indicate statistical significance. The data analyses were performed in SPSS, version 29 (IBM Corp) and R, version 4 (R Foundation for Statistical Computing, Vienna, Austria), and GraphPad Prism, version 10 (GraphPad Software, San Diego, CA).

## 3 Results

### 3.1 Baseline Characteristics of Study Participants

Of 1499 subjects with current asthma, the mean age (SD) was 49.5 (15.6) years and 61.6% were females (Supporting Information S1: Table E1). Eosinophilic asthma was present in 31.2% of subjects, while 55.5% had atopic asthma, 50.3% had T2‐high asthma, and 18.5% had atopic eosinophilic asthma. In addition, 20.8% had asthma with CRS, while 17.3% had asthma with nasal polyposis. Eosinophilic, atopic, and T2‐high asthma were more prevalent among males than females (Supporting Information S1: Table E2). In the subsample for NLF EDN, 386 subjects reported current asthma (Supporting Information S1: Table E1), of which 35.5% had eosinophilic and 67.1% had atopic asthma. The overlapping degree between T2‐high asthma and eosinophilic asthma was presented in Supporting Information S1: Figure E3.

### 3.2 EDN Levels in Eosinophilic Versus Non‐Eosinophilic Asthma

Those with eosinophilic asthma had significantly higher serum (62.5 [44.5–88.9] μg/L vs. 30.3 [21.8–42.6] μg/L) and NLF (14.1 [5.5–37.6] μg/L vs. 6.3 [2.7–12.7] μg/L) EDN levels than those with non‐eosinophilic asthma (Table 1). Also, NLF eosinophil percentage and FeNO levels were significantly higher in eosinophilic asthma (Table 1). These differences remained the same in males and females in sex‐stratified analyses.

**TABLE 1:** Serum and NLF biomarker levels in relation to eosinophilic asthma and atopic asthma.

	Subjects with current asthma									
	Non‐eosinophilic asthma		Eosinophilic asthma (blood eosinophil count [≥ 300 cells/mm^3^ ]		*p‐*value	Non‐atopic asthma		Atopic asthma		*p‐*value
	*N*	Median (Q1, Q3)	*N*	Median (Q1, Q3)		*N*	Median (Q1, Q3)	*N*	Median (Q1, Q3)	
Blood eosinophil count (cells/mm^3^)										
All participants	1032	125 (100–200)	467	400 (300–500)	**<** **0.001**	436	200 (100–300)	832	200 (100–300)	**0.022**
Males	370	150 (100–200)	206	400 (300–500)	**<** **0.001**	129	200 (100–300)	364	200 (100–300)	0.054
Females	662	110 (100–200)	261	400 (300–500)	**<** **0.001**	307	200 (100–280)	468	200 (100–300)	0.335
FeNO, ppb										
All participants	1028	17.1 (12.0–26.0)	466	26.9 (17.0–48.5)	**<** **0.001**	436	17.0 (11.6–24.6)	827	21.0 (14.0–35.0)	**<** **0.001**
Males	368	21.6 (14.7–32.5)	206	33.7 (20.2–57.3)	**<** **0.001**	129	20.0 (14.1–28.3)	362	26.8 (16.5–45.3)	**<** **0.001**
Females	660	15.4 (11.0–22.9)	260	23.8 (15.0–41.6)	**<** **0.001**	307	15.2 (10.9–22.6)	465	18.9 (12.1–27.0)	**<** **0.001**
Serum EDN levels (μg/L)										
All participants	1032	30.3 (21.8–42.6)	467	62.5 (44.5–88.9)	**<** **0.001**	436	34.3 (22.6–52.1)	832	37.1 (25.4–57.1)	**0.014**
Males	370	33.6 (24.4–46.6)	206	70.9 (51.3–99.4)	**<** **0.001**	129	36.9 (25.7–55.8)	364	42.1 (29.6–66.6)	0.051
Females	662	28.0 (20.6–40.2)	261	54.3 (39.7–80.0)	**<** **0.001**	307	33.1 (21.8–49.9)	468	33.5 (23.0–50.5)	0.513
NLF eosinophil percentage (%)										
All participants	249	0 (0–1.0)	137	1.0 (0–4.3)	**<** **0.001**	124	0 (0–0.5)	259	0.5 (0–3.0)	**<** **0.001**
Males	104	0.3 (0–1.5)	63	1.0 (0–4.0)	**0.005**	47	0 (0–0.5)	119	1.0 (0–2.5)	**<** **0.001**
Females	145	0 (0–0.5)	74	1.0 (0–5.0)	**<** **0.001**	77	0 (0–0)	140	0.5 (0–3.0)	**<** **0.001**
NLF EDN level (μg/L)										
All participants	249	6.3 (2.7–12.7)	137	14.1 (5.5–37.6)	**<** **0.001**	124	5.0 (2.0–10.4)	259	11.1 (4.5–23.8)	**<** **0.001**
Males	104	8.3 (4.3–19.2)	63	17.7 (8.7–43.6)	**<** **0.001**	47	7.6 (2.9–12.7)	119	13.9 (6.1–31.3)	**<** **0.001**
Females	145	5.2 (2.1–11.7)	74	11.6 (4.8–22.7)	**<** **0.001**	77	3.8 (0–9.3)	140	8.1 (3.4–16.8)	**<** **0.001**

*Note:* Mann‐Whitney *U* test was performed to compare biomarker levels in asthma phenotypes. Significant *p*‐values were shown in bold.

Abbreviations: EDN, eosinophil‐derived neurotoxin; FeNO, fractional exhaled nitric oxide; NLF, nasal lavage fluid; Q, quartile.

**FIGURE 1:**
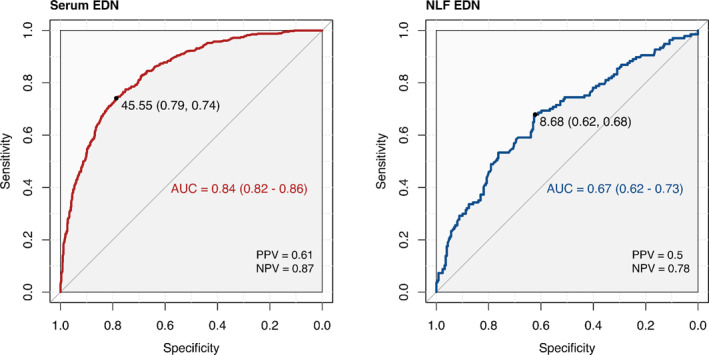
Receiver operating characteristic (ROC) curve of serum EDN level and NLF EDN level to distinguish eosinophilic asthma (blood eosinophil count ≥ 300 cells/mm^3^). AUC was presented as AUC with 95% CI in parenthesis. Optimal threshold values were calculated based on Youden index and presented with specificity and sensitivity in parenthesis. AUC, area under curve; EDN, eosinophil‐derived neurotoxin; NLF, nasal lavage fluid; NPV, negative predictive value; PPV, positive predictive value.

In ROC analysis, serum EDN yielded an AUC of 0.84 (0.82–0.86) in differentiating eosinophilic from non‐eosinophilic asthma (Figure 1). Sex‐stratified analyses yielded similar AUC value in males and females (Supporting Information S1: Table E3). On the other hand, NLF EDN had a lower AUC (0.67 [0.62–0.73]) (Figure 1). In sex‐stratified analysis for NLF EDN, AUC was 0.67 for males and 0.68 for females (Supporting Information S1: Table E3).

As a sensitivity analysis, ROC analyses were repeated for eosinophilic asthma using a threshold of ≥ 150 cells/mm^3^ for blood eosinophils (Supporting Information S1: Figure E4). Serum and NLF EDN gave similar AUC values (0.81 [0.78–0.83] and 0.67 [0.62–0.73], respectively) (Supporting Information S1: Figure E4).

Lastly, higher EDN levels were also associated with increased odds of eosinophilic asthma in adjusted models (Supporting Information S1: Table E4).

### 3.3 EDN Levels in Atopic Asthma Versus Non‐Atopic Asthma

Serum EDN levels were higher in those with atopic asthma than in those with non‐atopic asthma (37.1 [25.4–57.1] μg/L vs. 34.3 [22.6–52.1] μg/L), however, this finding did not remain significant in sex‐stratified analyses (Table 1). NLF EDN levels and NLF eosinophil percentages were also higher in subjects with atopic asthma than subjects with non‐atopic asthma in all participants, as well as among males and females separately (Table 1).

Both serum and NLF EDN did not yield an acceptable discrimination to differentiate atopic from non‐atopic asthma in ROC analysis (AUC 0.54 [0.51–0.58] and AUC 0.67 [0.61–0.73], respectively) (Supporting Information S1: Tables E5 and E6). This trend was also similar in males and females (Supporting Information S1: Tables E5 and E6).

### 3.4 EDN Levels in Atopic Eosinophilic Asthma Versus Non‐Atopic Eosinophilic Asthma

Both serum EDN levels and blood eosinophil counts were comparable between those with atopic eosinophilic and non‐atopic eosinophilic asthma (Table 2). On the other hand, NLF EDN levels (16.8 [7.8–45.1] μg/L vs. 9.0 [3.3–17.1] μg/L) and NLF eosinophil percentage (1.7 [0–5.5]% vs. 0 [0–1.3]%) were significantly higher in atopic eosinophilic than non‐atopic eosinophilic asthma, both in males and females (Table 2). NLF EDN had an AUC of 0.68 (0.58–0.77) to differentiate atopic eosinophilic asthma and non‐atopic eosinophilic asthma (Supporting Information S1: Table E6).

**TABLE 2:** Serum and NLF biomarker levels in those with atopic eosinophilic asthma compared to those with non‐atopic eosinophilic asthma.

	Non‐atopic eosinophilic asthma		Atopic eosinophilic asthma		*p‐*value
	*N*	Median (Q1, Q3)	*N*	Median (Q1, Q3)	
Blood eosinophil count (cells/mm^3^)					
All participants	112	400 (300–430)	277	400 (300–500)	0.678
Males	37	400 (300–480)	135	400 (300–500)	0.882
Females	75	400 (300–400)	142	400 (300–500)	0.613
FeNO, ppb					
All participants	112	21.0 (12.2–32.5)	276	28.5 (19.0–52.1)	**<** **0.001**
Males	37	26.4 (18.0–43.8)	135	37.3 (20.2–60.1)	0.055
Females	75	18.8 (11.0–28.3)	141	25.0 (17.6–42.7)	**<** **0.001**
Serum EDN levels (μg/L)					
All participants	112	60.4 (45.7–80.2)	277	59.5 (41.3–86.5)	0.831
Males	37	67.7 (53.2–88.5)	135	65.4 (47.0–95.1)	0.619
Females	75	53.7 (40.3–77.2)	142	52.5 (38.2–77.7)	0.992
NLF eosinophil percentage (%)					
All participants	41	0 (0–1.3)	95	1.7 (0–5.5)	**<** **0.001**
Males	16	0 (0–1.0)	47	1.5 (0–4.5)	**0.020**
Females	25	0 (0–1.5)	48	2.5 (0–9.5)	**0.002**
NLF EDN level (μg/L)					
All participants	41	9.0 (3.3–17.1)	95	16.8 (7.8–45.1)	**0.001**
Males	16	9.3 (7.1–18.6)	47	24.5 (8.7–58.7)	**0.047**
Females	25	7.6 (2.0–16.6)	48	12.6 (7.4–39.5)	**0.012**

*Note:* Mann‐Whitney *U* test was performed to compare biomarker levels in asthma phenotypes. Significant *p*‐values were shown in bold.

Abbreviations: EDN, eosinophil‐derived neurotoxin; FeNO, fractional exhaled nitric oxide; NLF, nasal lavage fluid; Q, quartile.

### 3.5 EDN Levels in T2‐High Versus T2‐Low Asthma

**FIGURE 2:**
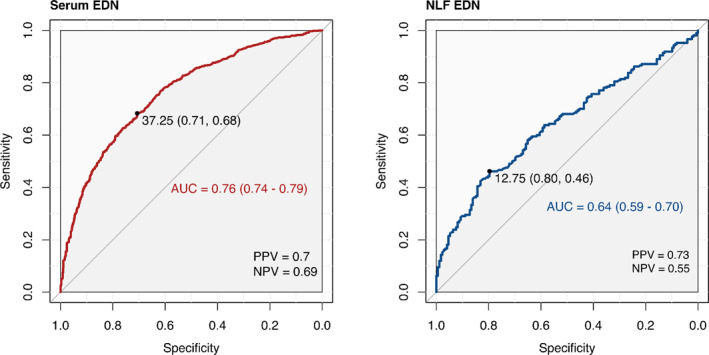
Receiver operating characteristic (ROC) curve for serum EDN level and NLF EDN level to distinguish T2‐high asthma (FeNO ≥ 25 ppb or blood eosinophil count ≥ 300 cells/mm^3^). AUC was presented as AUC with 95% CI in parenthesis. Optimal threshold values were calculated based on Youden index and presented with specificity and sensitivity in parenthesis. AUC, area under curve; EDN, eosinophil‐derived neurotoxin; NLF, nasal lavage fluid; NPV, negative predictive value; PPV, positive predictive value.

Subjects with T2‐high asthma had higher serum (49.7 [33.6–74.8] μg/L vs. 28.2 [20.9–40.3] μg/L) and NLF (11.2 [4.5–25.8] μg/L vs. 6.1 [2.5–12.3] μg/L) EDN levels than those with T2‐low asthma (Table 3). AUC was 0.76 (0.74–0.79) for serum EDN and 0.64 (0.59–0.70) for NLF EDN between these subgroups (Figure 2). Sex‐stratified ROC curves (Supporting Information S1: Table E3) indicated similar AUC values for both serum and NLF EDN in males and females.

**TABLE 3:** Serum and NLF biomarker levels in those with T2‐high asthma compared to those with T2‐low asthma.

	T2‐low asthma		T2‐high asthma		*p‐*value
	*N*	Median (Q1, Q3)	*N*	Median (Q1, Q3)	
Blood eosinophil count (cells/mm^3^)					
All participants	741	100 (100–200)	754	300 (200–400)	**<** **0.001**
Males	215	100 (100–200)	359	300 (200–400)	**<** **0.001**
Females	526	100 (100–200)	395	300 (200–400)	**<** **0.001**
FeNO, ppb					
All participants	741	14.0 (10.5–18.8)	753	31.5 (22.9–47.9)	**<** **0.001**
Males	215	16.0 (12.0–19.2)	359	34.0 (25.7–53.0)	**<** **0.001**
Females	526	13.8 (10.0–18.3)	394	28.5 (19.8–43.7)	**<** **0.001**
Serum EDN levels (μg/L)					
All participants	741	28.2 (20.9–40.3)	754	49.7 (33.6–74.8)	**<** **0.001**
Males	215	32.4 (24.0–45.3)	359	52.6 (34.5–77.4)	**<** **0.001**
Females	526	26.8 (20.0–38.0)	395	48.0 (32.9–70.7)	**<** **0.001**
NLF eosinophil percentage (%)					
All participants	172	0 (0–0.9)	210	0.5 (0–3.5)	**<** **0.001**
Males	59	0 (0–1.0)	106	1.0 (0–3.1)	**0.006**
Females	113	0 (0–0.5)	104	0 (0–4.0)	**<** **0.001**
NLF EDN level (μg/L)					
All participants	172	6.1 (2.5–12.3)	210	11.2 (4.5–25.8)	**<** **0.001**
Males	59	8.2 (4.6–16.2)	106	14.7 (5.1–35.4)	**0.011**
Females	113	4.6 (2.1–11.2)	104	9.1 (3.7–18.3)	**<** **0.001**

*Note:* Mann‐Whitney *U* test was performed to compare biomarker levels in asthma phenotypes. Significant *p*‐values were shown in bold.

Abbreviations: EDN, eosinophil‐derived neurotoxin; FeNO, fractional exhaled nitric oxide; NLF, nasal lavage fluid; Q, quartile.

In addition, we performed sensitivity analysis for T2‐high asthma, including presence of atopy in addition to blood eosinophil and/or FeNO. Similarly, serum and NLF EDN levels were significantly higher in T2‐high asthma with atopy (Supporting Information S1: Table E7). In ROC analyses, the AUC value of serum EDN was 0.68 (0.65–0.72), whereas the AUC value of NLF EDN was 0.69 (0.63–0.76) (Supporting Information S1: Tables E5 and E6).

### 3.6 EDN Levels in CRS, Nasal Polyposis, and CRSwNP

Serum and NLF EDN levels did not differ between subjects having asthma with versus without concomitant CRS (Supporting Information S1: Table E8). Similarly, blood eosinophil count, NLF eosinophil percentage, and FeNO levels were similar between both groups.

Subjects with nasal polyposis had higher serum (40.0 [27.8–62.5] μg/L vs. 36.6 [24.4–56.8] μg/L) and NLF (12.5 [5.8–24.2] μg/L vs. 8.0 [2.9–17.2] μg/L) EDN levels than subjects without nasal polyposis. Serum EDN level and blood eosinophil count did not differ between asthma with CRSwNP in comparison to asthma with CRSsNP (Supporting Information S1: Table E8). On the other hand, NLF EDN levels were higher in the asthma with CRSwNP group than asthma with CRSsNP group (13.3 [6.0–58.7] μg/L vs. 7.7 [2.4–14.1] μg/L). ROC analyses for serum and NLF EDN did not show an acceptable discrimination to differentiate asthma with CRS, nasal polyposis, or CRSwNP (Supporting Information S1: Tables E5 and E6).

### 3.7 EDN Levels in Obese Asthma

Serum and NLF EDN levels were comparable between those with non‐obese and obese asthma (Supporting Information S1: Table E9).

### 3.8 Comparison of EDN Levels in Relation to Other T2 Biomarkers

**FIGURE 3:**
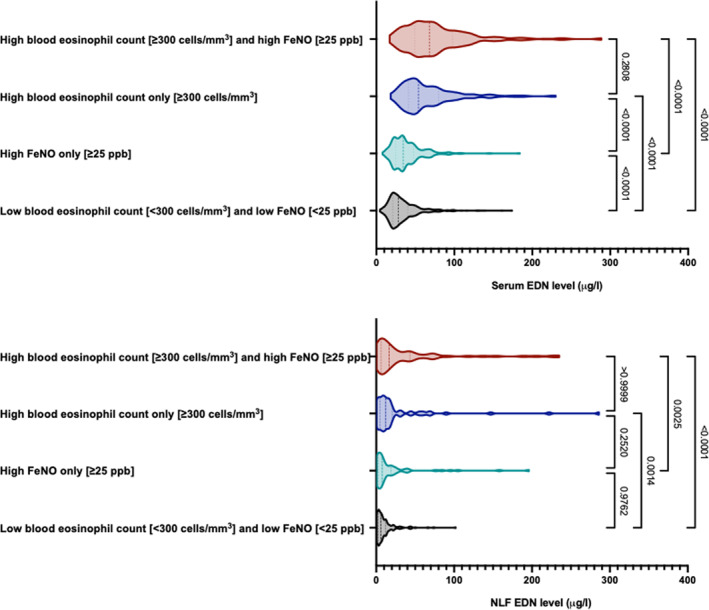
Serum and NLF EDN levels by low versus high blood eosinophil count and/or FeNO levels. Adjusted *p*‐values were calculated by Dunn's multiple comparison test. This analysis was performed in those with complete data with both for blood eosinophil count and FeNO. EDN, eosinophil‐derived neurotoxin; FeNO, fractional exhaled nitric oxide, NLF, nasal lavage fluid.

Both serum and NLF EDN levels were highest in those with high blood eosinophil and high FeNO levels (high eosinophil + FeNO group), followed by those with high‐eosinophil‐only, high‐FeNO‐only, and low eosinophil + FeNO, respectively (Figure 3). This finding remained the same in the sex‐stratified analysis (Supporting Information S1: Table E10). In multiple comparison tests, serum and NLF EDN levels of high eosinophil + FeNO group were comparable with the high‐eosinophil‐only group. Nevertheless, high eosinophil + FeNO group had significantly higher serum and NLF EDN compared to the high‐FeNO‐only group.

## 4 Discussion

### 4.1 Summary of Key Findings

Subjects with eosinophilic asthma had higher serum and NLF EDN levels than subjects with non‐eosinophilic asthma. ROC analyses suggested excellent discrimination for serum EDN to distinguish eosinophilic from non‐eosinophilic asthma and acceptable discrimination to distinguish T2‐high from T2‐low asthma, but this was not the case for NLF EDN. Serum and NLF EDN levels were also higher in those with atopic than those with non‐atopic asthma. Interestingly, only NLF, but not serum, EDN levels were significantly elevated in atopic eosinophilic asthma compared to non‐atopic eosinophilic asthma. EDN levels also differed in asthma phenotypes defined by the presence of nasal polyposis, but not by the presence of CRS or obesity. Subjects with T2‐high asthma had higher serum and NLF EDN levels. Both serum and NLF EDN levels were numerically highest in the high eosinophil + FeNO group, followed by the high‐eosinophil‐only, and high‐FeNO‐only groups.

### 4.2 Strengths and Limitations

To the best of our knowledge, this is the first study to quantify serum and NLF levels in adult asthma phenotypes in a population‐based setting. WSAS consists of general population‐representative adult cohorts providing generalizable findings to source asthma populations. In addition, EDN levels were measured in both nasal and blood samples, providing comprehensive data reflecting both local and systemic inflammation. On the other hand, it should be noted that cross‐sectional design of the current study does not imply any causal relationship. Therefore, current results should be interpreted with caution. Future longitudinal studies can also investigate EDN as a monitoring marker given the previous studies suggesting that EDN could reflect disease activity and monitor treatment response [27, 28, 29]. Specifically, in a post‐hoc analysis of the MENSA study, baseline EDN levels were able to predict treatment response to mepolizumab [29]. Accordingly, both treatment response and indicators of asthma control, including lung function and medication use, needs further investigation, particularly with longitudinal setting. Considering the applicability advantages of EDN, including the short‐ and long‐term stability and lack of circadian rhythm, it could provide additional benefits in comparison with blood eosinophil count [5, 30].

One of the main limitations of this study is the lack of bronchial or sputum samples, considered as the gold standard evaluation of eosinophilic asthma [31]. However, as a commonly used biomarker in both research and clinical settings, blood eosinophil count is a surrogate marker for sputum eosinophilia [32]. In addition, repeated measures design for blood eosinophil count is needed to address stability problems, as intrinsic and seasonal factors might lead to within‐subject variation [33]. The same factors might even affect sputum analysis. In a previous study, one in three subjects with mild‐to‐moderate asthma had intermittent sputum eosinophilia and approximately half of subjects had persistent non‐eosinophilic asthma [34], suggesting a possibility of false negative eosinophilic asthma in our study. It should be also noted that NLF eosinophil percentage was calculated within all cell types, not limited to leukocytes. In addition, EDN levels can be adjusted for albumin, total protein or exogenous markers in nasal samples in future studies to improve standardization and diagnostic accuracy [35, 36]. Lastly, as we previously discussed, information on the presence of CRS and nasal polyposis was collected through a self‐reported questionnaire [19]. The definition of CRS conceptualized based on the epidemiological definitions that were previously proposed [23]. However, clinical assessment of CRS and nasal polyposis with radiologic or endoscopic evaluation could provide a more objective assessment [24].

### 4.3 Comparison With Previous Findings

We previously revealed that current asthma was related to high serum EDN levels, but serum EDN showed a poor discriminatory performance in distinguishing current asthma in ROC analyses [19]. However, considering the heterogeneity in asthma, these findings might not be applicable to each recognized asthma phenotype. Therefore, in the current study, we examined the relation of EDN to different asthma phenotypes, particularly focusing on eosinophilic asthma. Not surprisingly, serum EDN yielded an AUC of 0.84 for eosinophilic asthma, while it did not show same performance to differentiate other asthma phenotypes. Atopy was also a factor related to high EDN in previous studies [7, 19, 37]. Although atopic asthma had higher serum and NLF EDN levels than non‐atopic asthma in our study, ROC analyses did not yield an acceptable discriminatory performance to distinguish these two phenotypes.

To date, only a few studies have investigated the role of EDN as biomarker in eosinophilic asthma [8, 9, 38, 39]. Our results are in line with previous findings using eosinophilic asthma/wheezing definitions based on sputum cell count [8, 38]. Those with eosinophilic asthma had higher serum EDN levels than mixed granulocytic and neutrophilic asthma in a previous study in adults [8]. Similarly, serum EDN levels were numerically higher in those with high sputum eosinophil count among children with recurrent wheezing, although this difference did not reach statistical significance [38]. In addition, these two studies were derived from hospital setting with a modest sample size [8, 38]. Our findings support previous literature by demonstrating that both serum and NLF EDN levels are higher in those with eosinophilic than in those with non‐eosinophilic asthma in a representative adult asthma sample.

Similar to our findings, a previous study has suggested that serum EDN is a predictor to differentiate asthma with blood eosinophilia (> 350 cells/mm^3^) in adults, with an AUC of 0.83 [9]. Our findings revealed similar AUC for serum EDN (0.84). Although NLF EDN was also higher in eosinophilic asthma, it showed poorer discrimination between eosinophilic and non‐eosinophilic asthma. These findings did not change notably when stratifying the analysis by sex. Interestingly, the optimal threshold values for both serum EDN and NLF EDN numerically differed between both sexes as males had higher threshold values. Our previous findings also indicated a significant difference between sex categories regardless of asthma and atopy status [19]. Cut‐off values obtained in the current study should be further confirmed in independent larger samples.

In our present study, those with atopic asthma had significantly higher serum and NLF EDN levels than those with non‐atopic asthma. Increased NLF EDN and nasal eosinophil percentage was also observed between atopic eosinophilic asthma and non‐atopic eosinophilic asthma. However, it was not the case for serum EDN levels. This finding was also consistent with a previous study on preschool wheeze suggesting no significant difference in urinary EDN levels between children with eosinophilia alone and those with eosinophilia and atopy [39]. A previous study suggested that nasal epithelium might have different Th2 gene expression from bronchial epithelium [40]. In addition, eosinophilic asthma definition was based on blood eosinophil count in the current study. Future studies with sputum or bronchoalveolar lavage eosinophil counts might provide a more robust comparison for nasal and serum EDN levels. Altogether, it can be argued that nasal samples might better reflect presence of atopy than serum samples; however further research is needed to confirm these findings.

Serum and NLF EDN levels were comparable between the asthma with CRS and the asthma without CRS groups in the present study. CRS is also a heterogenous condition where both T2 and non‐T2 inflammation pathways are implicated, hence it might need further investigation in detailed subgroups [16, 41]. A recent genome‐wide association study showed that there are both shared and distinct pathways between CRSwNP and asthma [42]. Interestingly, those with nasal polyposis had increased serum and NLF EDN levels, primarily in males. In line with our findings, nasal polyposis was also associated with high eosinophil count in adult‐onset asthma [43]. Nevertheless, the presence of CRS was defined based on self‐report of cardinal symptoms through a questionnaire; therefore, these findings should be interpreted carefully. Clinical evaluation of CRS and nasal polyposis with radiologic and endoscopic examination is needed to provide more robust evidence. Lastly, our sample size was insufficient to reach robust findings despite some preliminary results in these subgroups.

Our results reveal that those with T2‐high asthma have higher serum and NLF EDN levels than those with T2‐low asthma. After stratification based on FeNO and blood eosinophil counts in mutually exclusive groups, serum and NLF EDN levels were highest in the high eosinophil + FeNO group, followed by the high‐eosinophil‐only and the high‐FeNO‐only groups, respectively. Eosinophil levels are associated with IL‐5‐related pathways and FeNO reflects IL‐4/IL‐13‐related pathways [5]. It can be argued that eosinophil count and FeNO showed an additional effect on EDN levels. In a recent study, the high eosinophil + FeNO group demonstrated a better treatment response to anti‐IL‐5 treatments [44]. Considering the frequent overlap between both pathways, future studies should focus on clinical outcomes and treatment response in these subgroups.

## 5 Conclusion

Subjects with eosinophilic asthma, atopic asthma, T2‐high asthma, and asthma with nasal polyposis have elevated levels of serum and NLF EDN compared with those without these asthma phenotypes. However, only serum EDN appears to yield excellent discrimination between eosinophilic asthma and non‐eosinophilic asthma, as well as acceptable discrimination between T2‐high and T2‐low asthma.

## Author Contributions

**Saliha Selin Özuygur Ermis:** writing – original draft, formal analysis, conceptualization, investigation, visualization, writing – review and editing. **Carina Malmhall:** investigation, writing – review and editing, methodology, supervision. **Magnus P. Borres:** writing – review and editing. **Robert Moverare:** writing – review and editing. **Daniil Lisik:** writing – review and editing, data curation, formal analysis. **Reshed Abohalaka:** data curation, writing – review and editing. **Selin Ercan:** writing – review and editing, data curation. **Susanne Schmeisser:** methodology, project administration, resources, writing – review and editing. **Rani Basna:** data curation, formal analysis, methodology, writing – review and editing. **Roxana Mincheva:** writing – review and editing. **Goran Wennergren:** writing – review and editing, supervision. **Jan Lotvall:** project administration, writing – review and editing. **Linda Ekerljung:** writing – review and editing, data curation, investigation, project administration, resources. **Madeleine Radinger:** writing – review and editing, project administration. **Hannu Kankaanranta:** supervision, conceptualization, writing – review and editing, investigation, methodology, data curation, project administration, funding acquisition. **Bright I. Nwaru:** conceptualization, investigation, funding acquisition, writing – review and editing, formal analysis, supervision, resources, methodology, project administration, writing – original draft.

## Funding

VBG Group Herman Krefting Foundation for Asthma and Allergy Research, Sweden; Swedish Heart‐Lung Foundation (20210284, 244803506); Swedish Research Council (2019–00247); Swedish Asthma and Allergy Association (F2021‐0041), ALF agreement (ALFGBG‐966075, Grants from the Swedish state under the agreement between the Swedish Government and the county councils, Västra Götaland).

## Conflicts of Interest

Research kits were obtained from Thermo Fisher Scientific (Uppsala, Sweden)on behalf of West Sweden Asthma Study. Saliha Selin Özuygur Ermis reports conference attendance related fees from Thermo Fisher Scientific (Uppsala, Sweden). Robert Movérare is employed by Thermo Fisher Scientific (Uppsala, Sweden). Magnus P. Borres was employed by Thermo Fisher Scientific (Uppsala,Sweden). Hannu Kankaanranta reports fees for consultancies and lectures from AstraZeneca, Boehringer‐Ingelheim, Covis Pharma, GSK, Orion Pharma and Sanofi outside the current study. The remaining authors have nothing to disclose.

## Supporting information


Supporting Information S1

## Data Availability

Data are not publicly available due to privacy, legal or ethical restrictions.
