# Supplementary material for: Serum and Nasal Lavage Fluid Eosinophil‐Derived Neurotoxin Levels in Clinically Defined Asthma Phenotypes
Source: Clin Transl Allergy. 2026 Jun 23;16(6):e70183. doi: 10.1002/clt2.70183 (PMC13290645; doi:10.1002/clt2.70183)
Supplement: Supplementary file 1 — Supporting Information S1 [file CLT2-16-e70183-s001.docx]

**SUPPLEMENTARY MATERIAL**

**Serum and nasal lavage fluid eosinophil-derived neurotoxin levels in clinically defined asthma phenotypes**

Saliha Selin Özuygur Ermis^1^, Carina Malmhäll^1^, Magnus P. Borres^2,3^, Robert Movérare^3,4^, Daniil Lisik^1,5^, Reshed Abohalaka^1^, Selin Ercan^1^, Susanne Schmeisser^6^, Rani Basna^1,7^, Roxana Mincheva^1^, Göran Wennergren^8^, Jan Lötvall^1^, Linda Ekerljung^1,9^, Madeleine Rådinger^1^, Hannu Kankaanranta^1,10,11^, Bright I. Nwaru^1,12^

^1^Krefting Research Centre, Institute of Medicine, Sahlgrenska Academy, University of Gothenburg, Gothenburg, Sweden

^2^Department of Women’s and Children’s Health, Uppsala University, Uppsala, Sweden

^3^Thermo Fisher Scientific, Uppsala, Sweden

^4^Department of Medical Sciences: Respiratory, Allergy and Sleep Research, Uppsala University, Uppsala, Sweden

^5^Department of Public Health and Clinical Medicine, Section of Sustainable Health, The OLIN Unit, Umeå University, Umeå, Sweden

^6^Department of Clinical Immunology, Sahlgrenska University Hospital, Gothenburg, Sweden

^7^ Division of Geriatric Medicine, Department of Clinical Sciences in Malmö, Lund University, Malmö, Sweden

^8^Department of Paediatrics, Queen Silvia Children’s Hospital, Sahlgrenska University Hospital, University of Gothenburg, Gothenburg, Sweden

^9^Department of Respiratory Medicine and Allergology, Sahlgrenska University Hospital, Gothenburg, Sweden

^10^Tampere University Respiratory Research Group, Faculty of Medicine and Health Technology, Tampere University, Tampere, Finland

^11^Diagnostic Centre, The Wellbeing Services County of South Ostrobothnia, Seinäjoki, Finland

^12^Wallenberg Centre for Molecular and Translational Medicine, University of Gothenburg, Gothenburg, Sweden

Corresponding author

Saliha Selin Özuygur Ermis

Krefting Research Centre, Institute of Medicine, Sahlgrenska Academy

University of Gothenburg, Gothenburg, Sweden

Medicinaregatan 1F, Box 424, 405 30 Gothenburg, Sweden

[selin.ozuygur@gu.se](mailto:selin.ozuygur@gu.se)

**ORCID list of the authors:**

| Saliha Selin Özuygur Ermis | 0000-0003-3507-773X | selin.ozuygur@gu.se |
| --- | --- | --- |
| Carina Malmhäll | 0000-0001-6696-7570 | carina.malmhall@lungall.gu.se |
| Magnus P. Borres | 0000-0002-9045-2304 | magnus.borres@uu.se |
| Robert Movérare | 0000-0001-6611-5036 | robert.moverare@thermofisher.com |
| Daniil Lisik | 0000-0002-0220-5961 | daniil.lisik@gu.se |
| Reshed Abohalaka | 0000-0003-2803-2912 | reshed.abohalaka@gu.se |
| Selin Ercan | 0000-0002-9356-3042 | selin.ercan@gu.se |
| Susanne Schmeisser | N/A | susanne.schmeisser@vgregion.se |
| Rani Basna | 0000-0001-7510-8460 | rani.basna@gu.se |
| Roxana Mincheva | 0000-0002-6072-2748 | roxana.mincheva@gu.se |
| Göran Wennergren | 0000-0002-7010-7191 | goran.wennergren@pediat.gu.se |
| Jan Lötvall | 0000-0001-9195-9249 | jan.lotvall@gu.se |
| Linda Ekerljung | 0000-0001-5784-0041 | linda.ekerljung@lungall.gu.se |
| Madeleine Rådinger | 0000-0002-0652-7378 | madeleine.radinger@lungall.gu.se |
| Hannu Kankaanranta | 0000-0001-5258-0906 | hannu.kankaanranta@tuni.fi |
| Bright Ibeabughichi Nwaru | 0000-0002-2876-6089 | bright.nwaru@gu.se |

**Funding:** VBG Group Herman Krefting Foundation for Asthma and Allergy Research, Sweden; Swedish Heart‐Lung Foundation (20210284, 244803506); Swedish Research Council (2019-00247); Swedish Asthma and Allergy Association (F2021-0041), ALF agreement (ALFGBG-966075, Grants from the Swedish state under the agreement between the Swedish Government and the county councils, Västra Götaland).

**Conflict of interest statement:** Research kits were obtained from Thermo Fisher Scientific (Uppsala, Sweden) on behalf of West Sweden Asthma Study. Saliha Selin Özuygur Ermis reports conference attendance related fees from Thermo Fisher Scientific (Uppsala, Sweden). Robert Movérare is employed by Thermo Fisher Scientific (Uppsala, Sweden). Magnus P. Borres was employed by Thermo Fisher Scientific (Uppsala, Sweden). Hannu Kankaanranta reports fees for consultancies and lectures from AstraZeneca, Boehringer‐Ingelheim, Covis Pharma, GSK, Orion Pharma and Sanofi outside the current study. The remaining authors have nothing to disclose.

**INDEX**

1. **Supplementary material and method**
   1. **Definition of atopy**
   2. **Imputation of blood eosinophil count and FeNO levels**
   3. **Imputation of NLF data**
2. **Supplementary tables**

**Table E1.** Baseline characteristics and frequency of asthma phenotypes in the study population.

**Table E2.** Proportion of asthma phenotypes in males and females among those with current asthma.

**Table E3.** Estimates from receiver operating characteristic (ROC) analysis in males and females for serum and NLF EDN levels in distinguishing eosinophilic and T2-high asthma.

**Table E4.** Association between asthma phenotypes and EDN levels in adjusted models.

**Table E5.** Estimates from receiver operating characteristic (ROC) analysis for serum EDN levels in distinguishing asthma phenotypes

**Table E6**. Estimates from receiver operating characteristic (ROC) analysis for NLF EDN levels in distinguishing asthma phenotypes.

**Table E7.** Serum and NLF biomarker levels in those with T2-low asthma compared to those with T2-high asthma defined based on the presence of at least one following conditions: high blood eosinophil count, high FeNO or atopy.

**Table E8.** Serum and NLF biomarker levels in relation to asthma with CRS, nasal polyposis, and CRSwNP

**Table E9.** Serum and NLF biomarker levels in those with obese asthma compared to those without obese asthma.

**Table E10.** Serum and NLF EDN levels by low vs high blood eosinophil count and/or FeNO levels stratified in males and females.

1. **Supplementary figures**

**Figure E1.** Flow chart of study participants of those measured for serum EDN levels.

**Figure E2.** Flow chart of study participants of those measured for NLF EDN levels.

**Figure E3**. The Venn diagram for overlapping pattern between eosinophilic asthma (blood eosinophil count≥300 cells/mm^3^) and T2-high asthma (blood eosinophil count≥300 cells/mm^3^ or FeNO≥25 ppb). **Abbreviations.** EDN=eosinophil-derived neurotoxin, NLF=nasal lavage fluid, T2=type 2.

**Figure E4**. Receiver operating characteristic (ROC) curve for serum and NLF EDN levels to distinguish eosinophilic asthma defined based on blood eosinophil count ≥150 cells/mm^3^.

1. **Supplementary material and method**

**1.1) Definition of atopy**

Atopy was defined based on the presence of positive skin prick test (SPT) (≥3 mm) or specific immunoglobulin (IgE) positivity (≥0.35 kU_A_/L). SPT positivity was defined as a positive test to at least one of the following allergens: *dermatophagoides pteronyssinus*, *dermatophagoides farinae*, *alternaria alternata, cladosporium herbarum,* dog, cat, horse, timothy, mugwort, and birch. Specific IgE analyses were performed among those who had Phadiatop^TM^ positivity. These subjects were further analyzed, with specific IgE positivity defined as a positive test to at least one of the following allergens: *dermatophagoides pteronyssinus, dermatophagoides farinae,* cat, dog, horse, timothy*, cladosporium herbarum,* common silver birch, olive, mugwort, and wall pellitory. If Phadiatop^TM^ test was negative, these subjects were computed as non-atopic unless SPT was positive.

**1.2) Imputation of blood eosinophil count and FeNO levels**

Of the 2939 subjects measured for serum EDN, 1.4% of blood eosinophil data and 14.2% of FeNO data were missing, corresponding 1.5% and 12.0% in those with current asthma, respectively. Of the 878 subjects measured for NLF EDN, 1.1% of blood eosinophil data and 9.0% FeNO data were missing, corresponding 1.0% and 9.3% in those with current asthma.

As previously described, missing values were imputed by using multiple imputation by chained equations (MICE) with random forests (MICE-RF). This method was employed by using miceRanger R package. The mean of 100 imputed datasets were calculated for the analysis. Due to risk of misclassification in two datasets, imputed FeNO levels of 5 cases were excluded from the analysis.

- 1. **Imputation of NLF data**

Of the 878 subjects measured for NLF data, approximately 17% of the NLF samples were measured below the lower limit of normal, which corresponds 14% of the subjects with current asthma. For the imputation of this data, Gibbs-sampler approach was employed which was previously described elsewhere.

**References**

E1. Shah AD, Bartlett JW, Carpenter J, Nicholas O, Hemingway H. Comparison of random forest and parametric imputation models for imputing missing data using MICE: a CALIBER study. Am J Epidemiol. Mar 15 2014;179(6):764-74. doi:10.1093/aje/kwt312

E2. White IR, Royston P, Wood AM. Multiple imputation using chained equations: Issues and guidance for practice. Statistics in Medicine. 2011/02/20 2011;30(4):377-399. doi:<https://doi.org/10.1002/sim.4067>

E3. Özuygur Ermis SS, Malmhäll C, Borres MP, Movérare R, Lisik D, Abohalaka R, Ercan S, Schmeisser S, Basna R, Mincheva R, Wennergren G, Lötvall J, Ekerljung L, Rådinger M, Kankaanranta H, Nwaru BI. Serum and nasal lavage fluid eosinophil-derived neurotoxin levels and their determinants in adults. J Allergy Clin Immunol Glob. 2025 Jun 6;4(3):100510. doi: 10.1016/j.jacig.2025.100510. PMID: 40677614; PMCID: PMC12270069.

E4. Wei R, Wang J, Jia E, Chen T, Ni Y, Jia W. GSimp: A Gibbs sampler based left-censored missing value imputation approach for metabolomics studies. PLoS Comput Biol. 2018 Jan 31;14(1):e1005973. doi: 10.1371/journal.pcbi.1005973. PMID: 29385130; PMCID: PMC5809088.

1. **Supplementary tables**

**Table E1.** Baseline characteristics and frequency of asthma phenotypes in the study population.

| **General characteristics among those with current asthma** | **Those measured for serum EDN levels**  ***(N*=1,499)** | **Those measured for NLF EDN levels**  **(*n*=386)** |
| --- | --- | --- |
| **Sex, *n* (%)**  Male  Female | 576 (38.4)  923 (61.6) | 167 (43.3)  219 (56.7) |
| **Age, mean (SD)** | 49.5 (15.6) | 44.8 (15.7) |
| **BMI, mean (SD)** | 27.1 (4.9) | 26.8 (4.9) |
| **Asthma phenotypes among those with current asthma, *n* (%)** |  |  |
| **Based on blood eosinophilia (≥300 cells/mm^3^)** |  |  |
| Non-eosinophilic asthma | 1032 (68.8) | 249 (64.5) |
| Eosinophilic asthma | 467 (31.2) | 137 (35.5) |
| **Based on presence of atopy defined by SPT and/or IgE positivity** |  |  |
| Non-atopic asthma | 436 (29.1) | 124 (32.1) |
| Atopic asthma | 832 (55.5) | 259 (67.1) |
| Missing data | 231 (15.4) | 3 (0.8) |
| **Based on presence of atopy in those with blood eosinophilia (≥300 cells/mm^3^)** |  |  |
| Non-atopic eosinophilic asthma | 112 (7.5) | 41 (10.6) |
| Atopic eosinophilic asthma | 277 (18.5) | 95 (24.6) |
| Missing data | 78 (5.2) | 1 (0.3) |
| **Based on presence of high FeNO (≥25 ppb) or high blood eosinophil count (≥300 cells/mm^3^)** |  |  |
| T2-low asthma | 741 (49.4) | 172 (44.6) |
| T2-high asthma | 754 (50.3) | 210 (54.4) |
| Missing data | 4 (0.3) | 4 (1.0) |
| **Based on presence of high FeNO (≥25 ppb) or high blood eosinophil count (≥300 cells/mm^3^) or atopy** |  |  |
| T2-low asthma | 262 (17.5) | 62 (16.1) |
| T2-high asthma with atopy | 1133 (75.6) | 323 (83.7) |
| Missing data | 104 (6.9) | 1 (0.3) |
| **Based on presence of CRS** |  |  |
| Asthma without CRS | 1162 (77.5) | 298 (77.2) |
| Asthma with CRS | 312 (20.8) | 84 (21.8) |
| Missing data | 25 (1.7) | 4 (1.0) |
| **Based on presence of nasal polyposis** |  |  |
| Asthma without nasal polyposis | 990 (66.0) | 240 (62.2) |
| Asthma with nasal polyposis | 259 (17.3) | 68 (17.6) |
| Missing data | 250 (16.7) | 78 (20.2) |
| **Based on presence nasal polyposis in those with CRS** |  |  |
| Asthma with CRSsNP | 167 (11.1) | 43 (11.1) |
| Asthma with CRSwNP | 90 (6.0) | 27 (7.0) |
| Missing data | 55 (3.7) | 14 (3.6) |
| **Based on presence of obesity (BMI ≥30 kg/m^2^)** |  |  |
| Non-obese asthma | 1122 (74.8) | 295 (76.4) |
| Obese asthma | 363 (24.2) | 91 (23.6) |
| Missing data | 14 (0.9) | - |

**Abbreviations.** BMI=body mass index, CRS=chronic rhinosinusitis. CRSsNP=chronic rhinosinusitis without nasal polyposis, CRSwNP=chronic rhinosinusitis with nasal polyposis, EDN=eosinophil-derived neurotoxin, FeNO=fractional exhaled nitric oxide, IgE=immunoglobulin E, NLF=nasal lavage fluid, SD=standard deviation, SPT=skin prick test.

**Table E2.** Proportion of asthma phenotypes in males and females among those with current asthma.

| **Asthma phenotypes among those with current asthma** | **Those measured for serum EDN levels**  **(*N*=1,499)** | | | **Those measured for NLF EDN levels**  **(*n*=386)** | | |
| --- | --- | --- | --- | --- | --- | --- |
|  | **Males**  ***n* (%)** | **Females**  ***n* (%)** | ***p*-value** | **Males**  ***n* (%)** | **Females**  ***n* (%)** | ***p*-value** |
| **Based on blood eosinophilia (≥300 cells/mm^3^)** |  |  |  |  |  |  |
| Non-eosinophilic asthma | 370 (64.2) | 662 (71.7) | **0.002** | 104 (62.3) | 145 (66.2) | 0.423 |
| Eosinophilic asthma | 206 (35.8) | 261 (28.3) |  | 63 (37.7) | 74 (33.8) |  |
| **Based on presence of atopy defined by SPT and/or IgE positivity** |  |  |  |  |  |  |
| Non-atopic asthma | 129 (26.2) | 307 (39.6) | **<0.001** | 47 (28.3) | 77 (35.5) | 0.137 |
| Atopic asthma | 364 (73.8) | 468 (60.4) |  | 119 (71.7) | 140 (64.5) |  |
| **Based on presence of atopy in those with blood eosinophilia (≥300 cells/mm^3^)** |  |  |  |  |  |  |
| Non-atopic eosinophilic asthma | 37 (21.5) | 75 (34.6) | **0.005** | 16 (25.4) | 25 (34.2) | 0.262 |
| Atopic eosinophilic asthma | 135 (78.5) | 142 (65.4) |  | 47(74.6) | 48 (65.8) |  |
| **Based on presence of high FeNO (≥25 ppb) or high blood eosinophil count (≥300 cells/mm^3^)** |  |  |  |  |  |  |
| T2-low asthma | 215 (37.5) | 526 (57.1) | **<0.001** | 59 (35.8) | 113 (52.1) | **0.001** |
| T2-high asthma | 359 (62.5) | 395 (42.9) |  | 106 (64.2) | 104 (47.9) |  |
| **Based on presence of high FeNO (≥25 ppb) or high blood eosinophil count (≥300 cells/mm^3^) or atopy** |  |  |  |  |  |  |
| T2-low asthma | 65 (11.9) | 197 (23.1) | **<0.001** | 22 (13.2) | 40 (18.3) | 0.171 |
| T2-high asthma with atopy | 479 (88.1) | 654 (76.9) |  | 145 (86.8) | 178 (81.7) |  |
| **Based on presence of CRS** |  |  |  |  |  |  |
| Asthma without CRS | 449 (79.3) | 713 (78.5) | 0.713 | 127 (77.4) | 171 (78.4) | 0.815 |
| Asthma with CRS | 117 (20.7) | 195 (21.5) |  | 37 (22.6) | 47 (21.6) |  |
| **Based on presence of nasal polyposis** |  |  |  |  |  |  |
| Asthma without nasal polyposis | 362 (78.2) | 628 (79.9) | 0.471 | 92 (74.8) | 148 (80.0) | 0.281 |
| Asthma with nasal polyposis | 101 (21.8) | 158 (20.1) |  | 31 (25.2) | 37 (20.0) |  |
| **Based on presence nasal polyposis in those with CRS** |  |  |  |  |  |  |
| Asthma with CRSsNP | 53 (55.8) | 114 (70.4) | **0.018** | 14 (48.3) | 29 (70.7) | 0.057 |
| Asthma with CRSwNP | 42 (44.2) | 48 (29.6) |  | 15 (51.7) | 12 (29.3) |  |
| **Based on presence of obesity (BMI ≥30 kg/m^2^)** |  |  |  |  |  |  |
| Non-obese asthma | 430 (75.6) | 692 (75.5) | 0.991 | 120 (71.9) | 175 (79.9) | 0.065 |
| Obese asthma | 139 (24.4) | 224 (24.5) |  | 47 (28.1) | 44 (20.1) |  |

**Note.** Percentages were calculated based on columns. **Abbreviations.** BMI=body mass index, CRS=chronic rhinosinusitis, CRSsNP=chronic rhinosinusitis without nasal polyposis, CRSwNP= chronic rhinosinusitis with nasal polyposis, EDN=eosinophil-derived neurotoxin, FeNO=fractional exhaled nitric oxide, IgE= immunoglobulin E, NLF=nasal lavage fluid, SPT= skin orick test, T2=type 2.

**Table E3.** Estimates from receiver operating characteristic (ROC) analysis in males and females for serum and NLF EDN levels in distinguishing eosinophilic asthma (blood

eosinophil count ≥300 cells/mm^3^) and T2-high asthma (FeNO≥25 ppb or blood eosinophil count≥300 cells/mm^3^).

|  | Serum EDN | | | | | |
| --- | --- | --- | --- | --- | --- | --- |
|  | **AUC (95% CI)** | **Threshold based on Youden index (μg/L)** | **Sensitivity** | **Specificity** | **PPV** | **NPV** |
| Males |  |  |  |  |  |  |
| Eosinophilic asthma | 0.84 (0.81-0.88) | 51.85 | 0.75 | 0.83 | 0.71 | 0.86 |
| T2-high asthma | 0.73 (0.68-0.77) | 45.65 | 0.59 | 0.77 | 0.81 | 0.53 |
| Females |  |  |  |  |  |  |
| Eosinophilic asthma | 0.84 (0.81-0.86) | 37.25 | 0.81 | 0.71 | 0.52 | 0.9 |
| T2-high asthma | 0.77 (0.74-0.80) | 37.25 | 0.68 | 0.74 | 0.66 | 0.75 |
| NLF EDN | | | | | | |
| Males |  |  |  |  |  |  |
| Eosinophilic asthma | 0.67 (0.58-0.76) | 12.85 | 0.63 | 0.69 | 0.56 | 0.76 |
| T2-high asthma | 0.62 (0.53-0.70) | 16.55 | 0.47 | 0.78 | 0.79 | 0.45 |
| Females |  |  |  |  |  |  |
| Eosinophilic asthma | 0.68 (0.60-0.75) | 8.11 | 0.64 | 0.69 | 0.51 | 0.79 |
| T2-high asthma | 0.64 (0.57-0.72) | 6.66 | 0.63 | 0.63 | 0.61 | 0.65 |

**Abbreviations.** AUC=area under curve, CI=confidence interval, EDN=eosinophil-derived neurotoxin, PPV=positive predictive value, NLF=nasal lavage fluid, NPV=negative predictive value, T2=type 2.

**Table E4.** Association between asthma phenotypes and EDN levels in adjusted models.

|  | **Serum EDN**  **AOR (95% CI)** | | | | | **NLF EDN**  **AOR (95% CI)** | | | | |
| --- | --- | --- | --- | --- | --- | --- | --- | --- | --- | --- |
|  | Q1 | Q2 | Q3 | Q4 | Overall  p-value | Q1 | Q2 | Q3 | Q4 | Overall p-value |
| **Eosinophilic asthma*** | 1.0 | 4.33 (2.40-7.80) | 13.34 (7.62-23.38) | 56.0 (31.77-98.71) | <0.001 | 1.0 | 1.35 (0.67-2.71) | 3.62 (1.86-7.07) | 5.68 (2.85-11.30) | <0.001 |
| **T2-high asthma*** | 1.0 | 2.10 (1.52-2.90) | 4.18 (3.03-5.77) | 13.55 (9.44-19.45) | <0.001 | 1.0 | 1.14 (0.63-2.04) | 1.72 (0.94-3.12) | 3.67 (1.92-7.01) | <0.001 |
| **T2-high asthma with atopy** | 1.0 | 1.38 (0.98-1.96) | 2.56 (1.74-3.77) | 6.76 (4.10-11.14) | <0.001 | 1.0 | 0.99 (0.50-1.97) | 3.09 (1.34-7.11) | 5.07 (1.79-14.35) | 0.001 |
| **Atopic asthma*** | 1.0 | 1.16 (0.82-1.64) | 1.36 (0.96-1.94) | 1.49 (1.03-2.14) | 0.143 | 1.0 | 1.07 (0.56-2.05) | 2.28 (1.14-4.56) | 3.49 (1.63-7.45) | 0.002 |
| **Eosinophilic atopic asthma*** | 1.0 | 1.90 (0.43-8.37) | 1.25 (0.32-4.93) | 1.13 (0.29-4.38) | 0.641 | 1.0 | 1.39 (0.34-5.74) | 1.40 (0.41-4.77) | 3.13 (0.85-11.51) | 0.310 |
| **Asthma with CRS*** | 1.0 | 0.80 (0.55-1.14) | 0.86 (0.60-1.23) | 1.04 (0.73-1.49) | 0.420 | 1.0 | 0.69 (0.33-1.42) | 1.22 (0.62-2.43) | 1.13 (0.56-2.29) | 0.431 |
| **Asthma with nasal polyposis*** | 1.0 | 1.37 (0.90-2.06) | 1.51 (1.00-2.27) | 1.40 (0.92-2.11) | 0.235 | 1.0 | 1.45 (0.58-3.59) | 2.51 (1.08-5.87) | 2.80 (1.17-6.70) | 0.067 |
| **Asthma with CRSwNP*** | 1.0 | 1.61 (0.74-3.55) | 1.14 (0.52-2.46) | 1.53 (0.72-3.25) | 0.560 | 1.0 | 2.0 (0.30-13.22) | 2.51 (0.47-13.36) | 4.94 (0.84-28.94) | 0.359 |
| **Obese asthma**** | 1.0 | 1.15 (0.82-1.62) | 1.13 (0.81-1.59) | 0.98 (0.69-1.39) | 0.700 | 1.0 | 0.97 (0.50-1.90) | 0.52 (0.25-1.07) | 1.11 (0.56-2.21) | 0.173 |

**Note.** First quartile (Q1) was the referent. Models were adjusted for sex, age, body mass index, current smoking (*). Models were adjusted for sex, age, current smoking (**). **Abbreviations.** CRS= Chronic rhinosinusitis, CRSwNP= Chronic rhinosinusitis with nasal polyposis, EDN=Eosinophil-derived neurotoxin, NLF= nasal lavage fluid, T2= Type 2.

**Table E5.** Estimates from receiver operating characteristic (ROC) analysis for serum EDN levels in distinguishing asthma phenotypes.

|  | Serum EDN | | | | | |
| --- | --- | --- | --- | --- | --- | --- |
| All participants | **AUC (95% CI)** | **Threshold based on Youden index (μg/L)** | **Sensitivity** | **Specificity** | **PPV** | **NPV** |
| Atopic asthma | 0.54 (0.51-0.58) | 28.05 | 0.70 | 0.38 | 0.68 | 0.40 |
| Atopic eosinophilic asthma* | 0.49 (0.43-0.55) | 40.25 | 0.25 | 0.82 | 0.77 | 0.31 |
| T2-high asthma with atopy | 0.68 (0.65-0.72) | 37.25 | 0.56 | 0.74 | 0.90 | 0.28 |
| Asthma with CRS | 0.51 (0.47-0.55) | 76.70 | 0.17 | 0.88 | 0.27 | 0.80 |
| Asthma with nasal polyposis | 0.55 (0.51-0.59) | 29.15 | 0.74 | 0.36 | 0.23 | 0.84 |
| Asthma with CRSwNP | 0.56 (0.48-0.63) | 70.50 | 0.27 | 0.86 | 0.51 | 0.69 |
| Obese asthma | 0.50 (0.46-0.53) | 25.68 | 0.75 | 0.28 | 0.25 | 0.78 |
| Males |  |  |  |  |  |  |
| Atopic asthma | 0.56 (0.50-0.61) | 35.25 | 0.64 | 0.47 | 0.77 | 0.31 |
| Atopic eosinophilic asthma* | 0.53 (0.43-0.62) | 45.75 | 0.24 | 0.92 | 0.91 | 0.25 |
| T2-high asthma with atopy | 0.71 (0.65-0.77) | 45.65 | 0.51 | 0.86 | 0.96 | 0.19 |
| Asthma with CRS | 0.53 (0.48-0.59) | 95.65 | 0.16 | 0.90 | 0.30 | 0.81 |
| Asthma with nasal polyposis | 0.57 (0.51-0.64) | 72.85 | 0.33 | 0.81 | 0.32 | 0.81 |
| Asthma with CRSwNP | 0.57 (0.45-0.69) | 70.20 | 0.38 | 0.85 | 0.67 | 0.63 |
| Obese asthma* | 0.53 (0.47-0.58) | 69.25 | 0.84 | 0.25 | 0.27 | 0.83 |
| Females |  |  |  |  |  |  |
| Atopic asthma | 0.51 (0.47-0.56) | 26.45 | 0.67 | 0.38 | 0.62 | 0.43 |
| Atopic eosinophilic asthma* | 0.50 (0.42-0.58) | 42.75 | 0.32 | 0.75 | 0.70 | 0.37 |
| T2-high asthma with atopy | 0.65 (0.61-0.70) | 37.25 | 0.51 | 0.75 | 0.87 | 0.31 |
| Asthma with CRS | 0.49 (0.45-0.54) | 76.65 | 0.13 | 0.91 | 0.29 | 0.79 |
| Asthma with nasal polyposis | 0.53 (0.48-0.58) | 29.20 | 0.68 | 0.41 | 0.22 | 0.83 |
| Asthma with CRSwNP | 0.53 (0.43-0.63) | 44.45 | 0.44 | 0.66 | 0.35 | 0.74 |
| Obese asthma | 0.51 (0.47-0.55) | 27.45 | 0.69 | 0.38 | 0.26 | 0.79 |

**Abbreviations.** AUC=area under curve, CI=confidence interval, CRS=chronic rhinosinusitis, CRSsNP= chronic rhinosinusitis without nasal polyposis, CRSwNP=chronic rhinosinusitis with nasal polyposis, EDN=eosinophil-derived neurotoxin, PPV=positive predictive value, NPV=negative predictive value. ***Note.*** (*) Controls have higher EDN

levels compared to cases.

**Table E6.** Estimates from receiver operating characteristic (ROC) analysis for NLF EDN levels in distinguishing asthma phenotypes.

|  | NLF EDN | | | | | |
| --- | --- | --- | --- | --- | --- | --- |
| All participants | **AUC (95% CI)** | **Threshold based on Youden index (μg/L)** | **Sensitivity** | **Specificity** | **PPV** | **NPV** |
| Atopic asthma | 0.67 (0.61-0.73) | 9.67 | 0.54 | 0.74 | 0.81 | 0.43 |
| Atopic eosinophilic asthma | 0.68 (0.58-0.77) | 22.15 | 0.43 | 0.88 | 0.89 | 0.40 |
| T2-high asthma with atopy | 0.69 (0.63-0.76) | 10.45 | 0.48 | 0.85 | 0.95 | 0.24 |
| Asthma with CRS | 0.53 (0.46-0.60) | 34.70 | 0.23 | 0.89 | 0.36 | 0.80 |
| Asthma with nasal polyposis | 0.60 (0.52-0.67) | 11.75 | 0.56 | 0.63 | 0.30 | 0.83 |
| Asthma with CRSwNP | 0.64 (0.51-0.78) | 15.6 | 0.48 | 0.81 | 0.62 | 0.71 |
| Obese asthma* | 0.52 (0.45-0.59) | 5.25 | 0.44 | 0.67 | 0.29 | 0.80 |
| Males |  |  |  |  |  |  |
| Atopic asthma | 0.68 (0.59-0.77) | 10.35 | 0.61 | 0.72 | 0.85 | 0.43 |
| Atopic eosinophilic asthma | 0.67 (0.53-0.81) | 19.10 | 0.55 | 0.88 | 0.93 | 0.40 |
| T2-high asthma with atopy | 0.71 (0.61-0.81) | 10.35 | 0.57 | 0.86 | 0.97 | 0.23 |
| Asthma with CRS | 0.58 (0.46-0.70) | 34.10 | 0.41 | 0.89 | 0.52 | 0.84 |
| Asthma with nasal polyposis | 0.64 (0.53-0.76) | 11.75 | 0.71 | 0.60 | 0.37 | 0.86 |
| Asthma with CRSwNP | 0.66 (0.43-0.88) | 8.04 | 0.87 | 0.57 | 0.68 | 0.80 |
| Obese asthma | 0.49 (0.38-0.59) | 15.05 | 0.47 | 0.62 | 0.32 | 0.75 |
| Females |  |  |  |  |  |  |
| Atopic asthma | 0.66 (0.58-0.73) | 5.71 | 0.68 | 0.62 | 0.77 | 0.52 |
| Atopic eosinophilic asthma | 0.68 (0.55-0.81) | 5.51 | 0.85 | 0.48 | 0.76 | 0.63 |
| T2-high asthma with atopy | 0.67 (0.58-0.75) | 5.71 | 0.63 | 0.7 | 0.90 | 0.30 |
| Asthma with CRS | 0.51 (0.41-0.60) | 9.54 | 0.45 | 0.64 | 0.26 | 0.81 |
| Asthma with nasal polyposis | 0.56 (0.47-0.66) | 4.9 | 0.76 | 0.42 | 0.25 | 0.87 |
| Asthma with CRSwNP | 0.57 (0.37-0.77) | 15.90 | 0.33 | 0.86 | 0.50 | 0.76 |
| Obese asthma* | 0.56 (0.46-0.66) | 5.08 | 0.55 | 0.62 | 0.26 | 0.84 |

**Abbreviations.** AUC=area under curve, CI=confidence interval, CRS=chronic rhinosinusitis, CRSsNP=chronic rhinosinusitis without nasal polyposis, CRSwNP=chronic rhinosinusitis with nasal polyposis, EDN=eosinophil-derived neurotoxin, NLF=nasal lavage fluid, PPV=positive predictive value, NPV=negative predictive value. ***Note.*** (*)Controls

had higher EDN levels compared to cases.

**Table E7.** Serum and NLF biomarker levels in those with T2-low asthma compared to those with T2-high asthma defined based on the presence of at least one following conditions: high blood eosinophil count, high FeNO or atopy.

|  | T2-low asthma | | T2-high asthma  with atopy | |  |
| --- | --- | --- | --- | --- | --- |
| Blood eosinophil count (cells/mm^3^) | ***N*** | **Median**  **(Q1, Q3)** | ***N*** | **Median**  **(Q1, Q3)** | ***p-*value** |
| All participants | 262 | 130  (100-200) | 1133 | 200  (100-300) | **<0.001** |
| Males | 65 | 100  (100-200) | 479 | 200  (100-370) | **<0.001** |
| Females | 197 | 150  (100-200) | 654 | 200  (100-300) | **<0.001** |
| FeNO, ppb |  |  |  |  |  |
| All participants | 262 | 13.8  (10.1-18.0) | 1128 | 24.0 (14.8-38.0) | **<0.001** |
| Males | 65 | 16.0  (11.8-19.0) | 477 | 28.0  (17.5-45.4) | **<0.001** |
| Females | 197 | 13.5  (9.2-17.6) | 651 | 20.5  (13.1-32.2) | **<0.001** |
| Serum EDN levels (μg/L) |  |  |  |  |  |
| All participants | 262 | 27.5  (20.4-38.9) | 1133 | 40.3  (27.4-64.1) | **<0.001** |
| Males | 65 | 28.7  (22.5-40.5) | 479 | 46.2  (30.5-71.5) | **<0.001** |
| Females | 197 | 26.4  (19.7-38.6) | 654 | 37.6  (25.0-57.3) | **<0.001** |
| NLF eosinophil percentage (%) |  |  |  |  |  |
| All participants | 62 | 0  (0-0) | 323 | 0.5  (0-2.0) | **<0.001** |
| Males | 22 | 0  (0-0.6) | 145 | 0.5  (0-2.0) | **0.030** |
| Females | 40 | 0  (0-0) | 178 | 0  (0-2.5) | **<0.001** |
| NLF EDN levels (μg/L) |  |  |  |  |  |
| All participants | 62 | 4.1  (2.1-8.1) | 323 | 9.4  (3.9-20.3) | **<0.001** |
| Males | 22 | 5.8  (3.0-8.7) | 145 | 12.3  (5.6-25.3) | **0.001** |
| Females | 40 | 3.4  (0-7.9) | 178 | 7.8  (2.8-16.5) | **<0.001** |

**Note.** Mann-Whitney-U test was performed to compare biomarker levels in asthma phenotypes. **Abbreviations.** EDN=eosinophil-derived neurotoxin. FeNO=fractional exhaled nitric oxide, NLF=nasal lavage fluid, Q=quartile, T2= type 2.

**Table E8.** Serum and NLF biomarker levels in relation to asthma with CRS, nasal polyposis, and CRSwNP.

|  | Subjects with current asthma | | | | | | | | | | | | | | |
| --- | --- | --- | --- | --- | --- | --- | --- | --- | --- | --- | --- | --- | --- | --- | --- |
|  | **Asthma without CRS** | | **Asthma with CRS** | |  | **Asthma without nasal polyposis** | | **Asthma with nasal polyposis** | |  | **Asthma with CRSsNP** | | **Asthma with CRSwNP** | |  |
| Blood eosinophil count (cells/mm^3^) | ***N*** | **Median**  **(Q1, Q3)** | ***N*** | **Median**  **(Q1, Q3)** | ***p-*value** | ***N*** | **Median**  **(Q1, Q3)** | ***N*** | **Median**  **(Q1, Q3)** | ***p-*value** | ***N*** | **Median**  **(Q1, Q3)** | ***N*** | **Median**  **(Q1, Q3)** | ***p-***  **value** |
| All participants | 1162 | 200  (100-300) | 312 | 200  (100-300) | 0.968 | 990 | 200  (100-300) | 259 | 200  (100-390) | **<0.001** | 167 | 200  (100-300) | 90 | 200  (100-300) | 0.082 |
| Males | 449 | 200  (100-300) | 117 | 200  (100-300) | 0.432 | 362 | 200  (100-300) | 101 | 200  (100-400) | **0.012** | 53 | 200  (100-300) | 42 | 200  (100-500) | 0.370 |
| Females | 713 | 200  (100-300) | 195 | 200  (100-300) | 0.515 | 628 | 200  (100-300) | 158 | 200  (123-300) | **0.010** | 114 | 200  (100-300) | 48 | 200  (100-300) | 0.163 |
| FeNO, ppb |  |  |  |  |  |  |  |  |  |  |  |  |  |  |  |
| All participants | 1158 | 20.0  (13.3-32.2) | 311 | 19.0  (12.0-29.8) | 0.127 | 986 | 20.0  (13.0-32.1) | 258 | 21.0  (14.0-36.0) | **0.049** | 166 | 15.7  (11.0-27.6) | 90 | 20.8  (14.0-37.5) | **0.005** |
| Males | 447 | 24.6  (16.0-41.2) | 117 | 24.0  (15.6-36.4) | 0.546 | 360 | 25.0  (16.3-41.2) | 101 | 27.0  (17.3-51.6) | 0.168 | 53 | 22.0  (11.8-33.7) | 42 | 25.1  (17.8-44.3) | 0.104 |
| Females | 711 | 18.0  (12.0-26.3) | 194 | 15.7  (11.2-25.0) | 0.185 | 626 | 17.2  (11.8-26.3) | 157 | 19.0  (12.0-28.0) | 0.176 | 113 | 15.0 (11.0-24.3) | 48 | 18.8  (11.3-35.2) | 0.133 |
| Serum EDN level(μg/L) |  |  |  |  |  |  |  |  |  |  |  |  |  |  |  |
| All participants | 1162 | 36.4  (24.7-55.4) | 312 | 37.7  (23.9-59.8) | 0.594 | 990 | 36.6  (24.4-56.8) | 259 | 40.0  (27.8-62.5) | **0.020** | 167 | 37.3  (23.2-56.7) | 90 | 40.7  (25.7-72.0) | 0.131 |
| Males | 449 | 41.9  (28.1-65.9) | 117 | 45.4  (29.5-71.1) | 0.252 | 362 | 40.9  (28.3-65.7) | 101 | 48.0  (31.6-82.1) | **0.022** | 53 | 41.7  (28.3-62.1) | 42 | 42.8  (30.6-98.0) | 0.245 |
| Females | 713 | 33.5  (23.3-50.6) | 195 | 34.3  (21.5-53.5) | 0.800 | 628 | 34.0  (22.7-51.2) | 158 | 35.8  (24.5-53.3) | 0.265 | 114 | 35.1  (21.3-51.0) | 48 | 38.0  (21.6-56.2) | 0.554 |
| NLF eosinophil percentage (%) |  |  |  |  |  |  |  |  |  |  |  |  |  |  |  |
| All participants | 298 | 0  (0-1.5) | 84 | 0  (0-2.0) | 0.555 | 240 | 0  (0-2.0) | 68 | 0.5  (0-2.5) | 0.242 | 43 | 0  (0-1.5) | 27 | 1.0  (0-4.5) | 0.090 |
| Males | 127 | 0.5  (0-1.7) | 37 | 1.0  (0-4.3) | 0.209 | 92 | 0.5  (0-2.0) | 31 | 1.5  (0-4.5) | 0.125 | 14 | 0  (0-2.5) | 15 | 1.5  (0-5.0) | 0.205 |
| Females | 171 | 0  (0-1.5) | 47 | 0  (0-1.5) | 0.883 | 148 | 0  (0-2.0) | 37 | 0  (0-1.8) | 0.858 | 29 | 0  (0-1.3) | 12 | 0.3  (0-1.9) | 0.591 |
| NLF EDN level (μg/L) |  |  |  |  |  |  |  |  |  |  |  |  |  |  |  |
| All participants | 298 | 8.1  (3.3-17.7) | 84 | 9.0  (3.1-22.0) | 0.374 | 240 | 8.0  (2.9-17.2) | 68 | 12.5  (5.8-24.2) | **0.013** | 43 | 7.7  (2.4-14.1) | 27 | 13.3  (6.0-58.7) | **0.047** |
| Males | 127 | 10.2  (4.9-21.9) | 37 | 11.1  (4.4-65.8) | 0.150 | 92 | 9.0  (5.0-21.8) | 31 | 18.8  (8.4-43.2) | **0.017** | 14 | 7.8  (2.5-68.7) | 15 | 41.7  (8.4-70.4) | 0.150 |
| Females | 171 | 6.7  (2.8-14.9) | 47 | 6.7  (2.0-14.1) | 0.876 | 148 | 6.8  (2.3-15.0) | 37 | 9.3  (4.5-16.6) | 0.247 | 29 | 7.6  (2.2-13.9) | 12 | 8.5  (3.6-20.3) | 0.483 |

**Note.** Mann-Whitney U *t*est was performed to compare biomarker levels in asthma phenotypes. **Abbreviations.** CRS = chronic rhinosinusitis, CRSsNP = chronic rhinosinusitis without nasal polyposis, CRSwNP = chronic rhinosinusitis with nasal polyposis, EDN = eosinophil-derived neurotoxin, FeNO = fractional exhaled nitric oxide, NLF = nasal lavage fluid, Q = quartile.

**Table E9.** Serum and NLF biomarker levels in those with obese asthma compared to those without obese asthma.

|  | Non-obese asthma | | Obese asthma | |  |
| --- | --- | --- | --- | --- | --- |
| Blood eosinophil count (cells/mm^3^) | ***N*** | **Median**  **(Q1, Q3)** | ***N*** | **Median**  **(Q1, Q3)** | ***p-*value** |
| All participants | 1122 | 200  (100-300) | 363 | 200  (100-300) | 0.963 |
| Males | 430 | 200  (100-300) | 139 | 200  (100-300) | 0.426 |
| Females | 692 | 200  (100-300) | 224 | 200  (100-300) | 0.561 |
| FeNO, ppb |  |  |  |  |  |
| All participants | 1119 | 20.0  (13.0-33.1) | 361 | 18.7  (12.3-28.0) | **0.035** |
| Males | 428 | 24.6  (16.2-41.0) | 139 | 23.3  (15.0-34.0) | 0.201 |
| Females | 691 | 18.0  (12.0-27.0) | 222 | 17.0  (11.6-23.0) | 0.091 |
| Serum EDN levels (μg/L) |  |  |  |  |  |
| All participants | 1122 | 36.4  (24.2-57.4) | 363 | 36.6  (25.8-55.5) | 0.907 |
| Males | 430 | 42.1  (29.0-69.1) | 139 | 41.2  (27.6-62.1) | 0.363 |
| Females | 692 | 33.2  (22.4-51.0) | 224 | 34.4  (23.6-49.9) | 0.624 |
| NLF eosinophil percentage (%) |  |  |  |  |  |
| All participants | 295 | 0  (0-1.7) | 91 | 0  (0-1.5) | 0.867 |
| Males | 120 | 0.5  (0-2.0) | 47 | 0  (0-1.5) | 0.300 |
| Females | 175 | 0  (0-1.5) | 44 | 0  (0-2.0) | 0.602 |
| NLF EDN levels (μg/L) |  |  |  |  |  |
| All participants | 295 | 8.6  (3.4-17.3) | 91 | 7.3  (2.8-19.8) | 0.503 |
| Males | 120 | 10.5  (5.5-24.2) | 47 | 11.1  (3.4-24.5) | 0.765 |
| Females | 175 | 7.5  (2.8-14.1) | 44 | 4.6  (0.5-15.0) | 0.220 |

**Note.** Mann-Whitney U test was performed to compare biomarker levels in asthma phenotypes. **Abbreviations.** EDN = eosinophil-derived neurotoxin. FeNO = fractional exhaled nitric oxide, NLF = nasal lavage fluid, Q = quartile.

**Table E10.** Serum and NLF EDN levels by low vs high blood eosinophil count and/or FeNO levels stratified in males and females.

|  | Subjects with current asthma | | | | | | | | |
| --- | --- | --- | --- | --- | --- | --- | --- | --- | --- |
|  | **All participants** | | | **Males** | | | **Females** | | |
| Serum EDN levels (µg/l) | ***N*** | **Median**  **(Q1, Q3)** | ***p-*value** | ***N*** | **Median**  **(Q1, Q3)** | ***p-*value** | ***N*** | **Median**  **(Q1, Q3)** | ***p-*value** |
| Low blood eosinophil count [<300 cells/mm^3^] and low FeNO [<25 ppb] | 741 | 28.2  (20.9-40.3) | **<0.001** | 215 | 32.4  (24.0-45.3) | **<0.001** | 526 | 26.8  (20.0-38.0) | **<0.001** |
| High FeNO only  [≥25 ppb] | 287 | 34.4  (24.8-47.7) |  | 153 | 35.1  (26.4-48.2) |  | 134 | 33.3  (23.3-45.1) |  |
| High blood eosinophil count  only  [≥300 cells/mm3] | 216 | 54.0  (41.2-80.0) |  | 77 | 65.7  (50.6-90.8) |  | 139 | 50.4  (37.3-74.9) |  |
| High blood eosinophil count [≥300 cells/mm^3^] and high FeNO [≥25 ppb] | 250 | 68.1  (49.3-97.6) |  | 129 | 74.0  (51.8-101.0) |  | 121 | 64.9  (47.9-88.9) |  |
| NLF EDN levels (µg/l) | ***N*** | **Median**  **(Q1, Q3)** | ***p-*value** | ***N*** | **Median**  **(Q1, Q3)** | ***p-*value** | ***N*** | **Median**  **(Q1, Q3)** | ***p-*value** |
| Low blood eosinophil count [<300 cells/mm^3^] and low FeNO [<25 ppb] | 172 | 6.1  (2.5-12.3) | **<0.001** | 59 | 8.2  (4.6-16.2) | **0.002** | 113 | 4.6  (2.1-11.2) | **<0.001** |
| High FeNO only  [≥25 ppb] | 73 | 7.6  (2.8-19.1) |  | 43 | 9.8  (4.1-22.4) |  | 30 | 5.9  (0-15.4) |  |
| High blood eosinophil count  only  [≥300 cells/mm^3^] | 61 | 12.2  (5.0-20.8) |  | 24 | 15.9  (7.1-40.2) |  | 37 | 11.3  (3.9-18.7) |  |
| High blood eosinophil count [≥300 cells/mm^3^] and high FeNO [≥25 ppb] | 75 | 16.8  (6.4-43.2) |  | 39 | 23.8  (8.7-56.4) |  | 36 | 11.8  (5.9-39.1) |  |

**Note.** Kruskal-Wallis test was performed to compare serum and NLF EDN levels. **Abbreviations.** EDN = eosinophil-derived neurotoxin, FeNO= fractional exhaled nitric oxide, NLF = nasal lavage fluid, Q = quartile. This analysis was performed in those with complete data with both for blood eosinophil count and FeNO.

1. **Supplementary figures**

**Figure E1.** Flow chart of study participants of those measured for serum EDN levels. ***Abbreviations.*** EDN=eosinophil-derived neurotoxin, WSAS=West Sweden Asthma Study. ***Note.*** (*) 22 subjects were excluded for duplicated visit.

**Figure E2.** Flow chart of study participants of those measured for NLF EDN levels. ***Abbreviations.*** EDN=eosinophil-derived neurotoxin, NLF=nasal lavage fluid, WSAS=West Sweden Asthma Study.


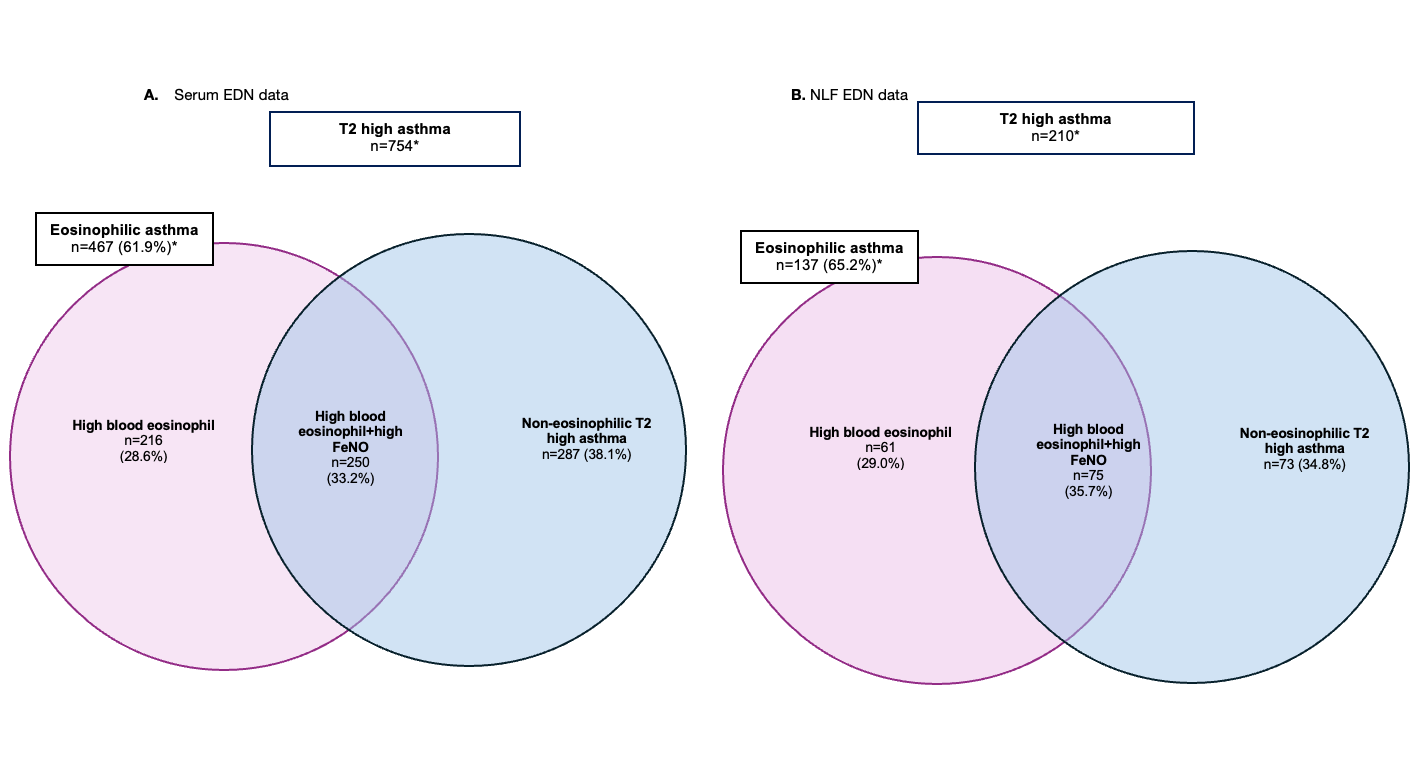


**Figure E3**. The Venn diagram for overlapping pattern between eosinophilic asthma (blood eosinophil count≥300 cells/mm^3^) and T2-high asthma (blood eosinophil count≥300 cells/mm^3^ or FeNO≥25 ppb). **Note.** Since Venn diagrams demonstrate those with complete data for both blood eosinophil count and FeNO, one subjects with T2-high asthma could not be demonstrated due to lack of FeNO data despite having high blood eosinophil count. **Abbreviations.** EDN=eosinophil-derived neurotoxin, NLF=nasal lavage fluid, T2=type 2.

**Figure E4.** Receiver operating characteristic (ROC) curve for serum and NLF EDN levels to distinguish eosinophilic asthma defined based on blood eosinophil count ≥150 cells/mm^3^. **Abbreviations.** AUC=area under curve, EDN=eosinophil-derived neurotoxin, NLF=nasal lavage fluid, NPV=negative predictive value, PPV=positive predictive value. ***Note.*** AUC was presented as AUC with 95% CI in parenthesis. Optimal threshold values were calculated based on Youden index and presented with specificity and sensitivity in parenthesis.
